# Antibiotic Use Among Hospitalized Children and Neonates in China: Results From Quarterly Point Prevalence Surveys in 2019

**DOI:** 10.3389/fphar.2021.601561

**Published:** 2021-03-29

**Authors:** Chu-ning Wang, Jianning Tong, Bin Yi, Benedikt D. Huttner, Yibing Cheng, Shuangjie Li, Chaomin Wan, Qingxiong Zhu, Qionghua Zhou, Shiyong Zhao, Zhiqiang Zhuo, Daobin Wang, Chunmei Jia, Qing-wen Shan, Yun Zhao, Chenfu Lan, Dongchi Zhao, Yibo Zhou, Jing Liu, Chunhui Zhu, Yu Zhu, Rui Li, Xiaodan Wu, Zhenghong Qi, Caihong Wang, Huiling Gao, Wenyu Ye, Liling Zhang, Xiaohong Xu, Hui Hu, Pu Yang, Nicola Magrini, Mei Zeng

**Affiliations:** ^1^ Department of Infectious Diseases, Children’s Hospital of Fudan University, Shanghai, China; ^2^ Department of Pediatric Gastroenterology and Infectious Diseases, Qingdao Women and Children’s Hospital, Qingdao, China; ^3^ Department of Neonatology, Gansu Provincial Maternity and Child-care Hospital, Lanzhou, China; ^4^ Division of Infectious Diseases, Geneva University Hospitals and Faculty of Medicine, University of Geneva, Geneva, Switzerland; ^5^ Department of Emergency, Children's Hospital Affiliated to Zhengzhou University (Henan Children's Hospital), Zhengzhou, China; ^6^ Department of Hepatology, Hunan Children’s Hospital, Changsha, China; ^7^ Department of Pediatrics, West China Second Hospital, Sichuan University, Chengdu, China; ^8^ Department of Infectious Diseases, Children’s Hospital of Jiangxi Province, Nanchang, China; ^9^ Department of Infectious Diseases, Hainan Women and Children’s Medical Center, Haikou, China; ^10^ Department of Infectious Diseases, Hangzhou Children’s Hospital, Hangzhou, China; ^11^ Department of Infectious Diseases, Xiamen Children’s Hospital, Xiamen, China; ^12^ Department of Pediatrics, People’s Hospital of Zhecheng County, Shangqiu, China; ^13^ Department of Pediatrics, The Fourth Hospital of Baotou, Baotou, China; ^14^ Department of Pediatrics, The First Affiliated Hospital of Guangxi Medical University, Nanning, China; ^15^ Department of Respiratory Medicine, Chengdu Children Special Hospital, Chengdu, China; ^16^ Department of Pediatrics, Lishui Maternal and Child Health Care Hospital, Lishui, China; ^17^ Department of Pediatrics, Zhongnan Hospital of Wuhan University, Wuhan, China; ^18^ Department of General Pediatrics, Children’s Hospital Affiliated to Zhengzhou University (Henan Children’s Hospital), Zhengzhou, China; ^19^ Department of Infectious Diseases, Hunan Children's Hospital, Changsha, China; ^20^ Department of Pharmacy, The Fourth Hospital of Baotou, Baotou, China; ^21^ Department of Digestive Infection, Gansu Provincial Maternity and Child-care Hospital, Lanzhou, China; ^22^ Department of Essential Medicines and Health Products, World Health Organization, Geneva, Switzerland

**Keywords:** pediatrics, inpatient, antibiotic, antimicrobial stewardship, Point prevalence survey, AWaRe classification

## Abstract

**Background:** Antimicrobial resistance is a significant clinical problem in pediatric practice in China. Surveillance of antibiotic use is one of the cornerstones to assess the quality of antibiotic use and plan and assess the impact of antibiotic stewardship interventions.

**Methods:** We carried out quarterly point prevalence surveys referring to WHO Methodology of Point Prevalence Survey in 16 Chinese general and children’s hospitals in 2019 to assess antibiotic use in pediatric inpatients based on the WHO AWaRe metrics and to detect potential problem areas. Data were retrieved via the hospital information systems on the second Monday of March, June, September and December. Antibiotic prescribing patterns were analyzed across and within diagnostic conditions and ward types according to WHO AWaRe metrics and Anatomical Therapeutic Chemical (ATC) Classification.

**Results:** A total of 22,327 hospitalized children were sampled, of which 14,757 (66.1%) were prescribed ≥1 antibiotic. Among the 3,936 sampled neonates (≤1 month), 59.2% (*n* = 2,331) were prescribed ≥1 antibiotic. A high percentage of combination antibiotic therapy was observed in PICUs (78.5%), pediatric medical wards (68.1%) and surgical wards (65.2%). For hospitalized children prescribed ≥1 antibiotic, the most common diagnosis on admission were lower respiratory tract infections (43.2%, *n* = 6,379). WHO Watch group antibiotics accounted for 70.4% of prescriptions (*n* = 12,915). The most prescribed antibiotic ATC classes were third-generation cephalosporins (41.9%, *n* = 7,679), followed by penicillins/β-lactamase inhibitors (16.1%, *n* = 2,962), macrolides (12.1%, *n* = 2,214) and carbapenems (7.7%, *n* = 1,331).

**Conclusion:** Based on these data, overuse of broad-spectrum Watch group antibiotics is common in Chinese pediatric inpatients. Specific interventions in the context of the national antimicrobial stewardship framework should aim to reduce the use of Watch antibiotics and routine surveillance of antibiotic use using WHO AWaRe metrics should be implemented.

## Introduction

Antimicrobial resistance has become a major public health problem, especially in low and middle income countries like China, where the burden of infectious diseases and the frequency of antibiotic use are very high. According to national surveillance data, the prevalence of extended-spectrum beta lactamase (ESBL)-producing Enterobacteriaceae is >50%. The carbapenem resistance in *Klebsiella pneumoniae* and *Acinetobacter baumannii* is 13.4 and 28.5% in children’s hospital, highlighting the growing challenge of treating pediatric infectious diseases in China (National Health Commission of the People’s Republic of China, 2018b).

Appropriate use of antibiotics is fundamental to slow the emergence and spread of antibiotic resistance and extend the useful lifetime of existing antibiotics (Goossens, 2009). In 2015, the World Health Assembly adopted a global action plan on antimicrobial resistance and highlighted surveillance of antibiotic use as one of its key strategies (World Health Organization, 2015). Since 2011, the Chinese government has initiated a series of national antimicrobial management strategies to supervise and restrict antibiotic use (Xiao and Li, 2013). These interventions have been associated with a reduction in overall antibiotic prescribing, but the prevalence of infections with antibiotic-resistant WHO priority pathogens remains high in China (National Health Commission of the People’s Republic of China, 2018b). Children are high users of antibiotics, but few surveillance data truly reflect the current situation of antibiotic use in Chinese pediatric patients because the methodology and the indicators (such as defined daily doses) adopted in the existing national or hospital antibiotic surveillance program are not suitable for children. According to national surveillance data, antibiotic prescribing rate was 36.7% in 2017 (National Health Commission of the People’s Republic of China, 2018b). However, pediatric data are not separated from adult data for analysis in the national antibiotic surveillance protocol.

Recognizing the need for practical tools for antibiotic stewardship at the national and international levels to assess and supervise the appropriate use of antibiotics, WHO updated the Model List of Essential Medicines for Children (EMLc) in 2017 and grouped antibiotics into three main categories——Access, Watch, and Reserve (AWaRe) (WHO Expert Committee, 2017). Access group antibiotics have activity against a wide range of commonly encountered susceptible pathogens while showing relatively low resistance potential. Watch group antibiotics includes most of the highest priority agents among the Critically Important Antimicrobials (CIA) for Human Medicine. Reserve group antibiotics should be reserved for treatment of confirmed or suspected infections due to multi-drug-resistant organisms (WHO Expert Committee, 2017). In 2019, WHO extended AWaRe metrics to 177 antibiotics used across the globe (WHO Expert Committee on the Selection and Use of Essential Medicines, 2019; World Health Organization, 2019b), and a target indicator based on AWaRe was proposed by WHO which specifies a country level target of at least 60% of antibiotic consumption being from the ACCESS group (World Health Organization, 2018a). So far, most of the surveillance studies using WHO AWaRe metrics have been conducted by international research groups such as GARPEC and Global-PPS (Hsia et al., 2019a; Hsia et al., 2019b). Our previous study showed that WHO WATCH group antibiotics accounted for 82.2% of all antibiotic therapies in pediatric outpatients in China in 2018 (Wang et al., 2020). To date, only one multi-center point prevalence survey described antibiotic use among hospitalized children in China, which retrieved a 1-day cross-sectional survey in winter season and included a relatively small sample (Zhang et al., 2018). To further detect problem areas in pediatric antibiotic use in China, identify opportunities for improvement and strengthen evidence-based antimicrobial stewardship interventions in pediatric inpatient, we carried out this repeated point prevalence study to investigate antibiotic prescribing practices in Chinese pediatric inpatients and evaluate disease-based antibiotic prescribing based on the WHO AWaRe metrics (WHO Expert Committee on the Selection and Use of Essential Medicines, 2019).

## Results

### General Information of Sampled Hospitalized Children

Among the 16 participating hospitals, 13 hospitals conducted surveys on all four time points, three on March, June and September and one on March and June. A total of 23,651 neonatal and pediatric patients were hospitalized at 8 am on the days of surveys, of which, 1,324 (5.6%) were excluded based on the pre-specified inclusion/exclusion criteria and 22,327 (94.4%) were eligible for the final analysis. Of the eligible patients, 95.5% (*n* = 21,324) were from tertiary care hospitals and 4.5% (*n* = 1,003) were from secondary care hospitals; 92.0% (*n* = 20,535) were from specialized children’s hospitals and 8.0% (*n* = 1,792) were from pediatric wards of general hospitals. Of 22,327 pediatric inpatients, 17.6% were neonates (0 to <1 month) and 42.3% were children ≤1 year (Table 1).

**TABLE 1:** General information of sampled hospitalized children.

Hospitals	Number of inpatients (%)
Tertiary hospitals	
C1	1,599 (7.2)
C2	808 (3.6)
C3	1,923 (8.6)
C4	670 (3.0)
C5	3,559 (15.9)
C6	898 (4.0)
C7	4,381 (19.6)
C8	430 (1.9)
C10	4,603 (20.6)
C11	1,186 (5.3)
G1	462 (2.1)
G3	604 (2.7)
G5	201 (0.9)
Secondary hospitals	
S1	344 (1.5)
S3	134 (0.6)
S4	525 (2.4)
Ward types	
Pediatric medical wards	12,197 (54.6)
Pediatric surgical wards	3,278 (14.7)
High-risk wards	1,806 (8.1)
PICUs	838 (3.8)
Neonatal wards	3,186 (14.3)
NICUs	1,022 (4.6)
Age groups	
0 to <1 month	3,936 (17.6)
1 month to <1 year	5,506 (24.7)
1 year to <3 years	4,545 (20.4)
3 years to <7 years	5,101 (22.8)
7 years to <12 years	2,519 (11.3)
Sex	
Male	13,490 (60.4)
Female	8,837 (39.6)
Total	22,327

### Antibiotics Prescribing Patterns and Distributions

Of the 22,327 eligible hospitalized children, 14,757 (66.1%) received ≥1 antibiotic prescription, of which, 6,275 (42.5%) were neonates and 9,430 (63.9%) were children younger than 3 years. The percentage of children receiving antibiotic therapies varied by hospitals (median 70.3%, IQR 64.6–74.1%, range 36.8–94.2%) and age groups (median 63.5%, IQR 58.8%-69.0, range 55.0–71.6%). The percentage of patients receiving antibiotics was 66.2% (*n* = 8,926) and 66.0% (*n* = 5,831) in male and female inpatients. The percentage of patients receiving antibiotics was higher in secondary care hospitals (86.0%, range 64.9–94.2%) than in tertiary care hospitals (65.2%, median 69.8%, IQR 64.2–71.2%, range 36.8–89.6%); antibiotics were prescribed most frequently in children aged 1 month to <1 year (71.6%). Of the 14,757 patients in which antibiotics prescribed, percentages of inpatients receiving intravenous antibiotic therapy and combination antibiotic therapy ranged from 72.5 to 100% (median 97.4%, IQR 91.3–98.9%) and 7.8–41.8% (median 23.4%, IQR 16.8–27.3%) within the 16 participating hospitals, respectively (Table 2).

**TABLE 2:** Number (percentage) of inpatients in which combination antibiotic therapy was prescribed (Top 10).

Combination antibiotic therapy	Number (percentage) of inpatient in which combination antibiotic therapy was prescribed
Macrolides + third generation cephalosporins	868 (5.9%)
Other antibacterials (J01X) + third generation cephalosporins	537 (3.6%)
Carbapenems + other antibacterials (J01X)	404 (2.7%)
Macrolides + penicillins/β-lactamase inhibitors	350 (2.4%)
Other penicillins + third generation cephalosporins	295 (2.0%)
Penicillins/β-lactamase inhibitors + third generation cephalosporins	141 (1.0%)
Sulfonamids and trimethoprim + third generation cephalosporins	126 (0.9%)
Carbapenems + macrolides	84 (0.6%)
Macrolides + second generation cephalosporins	82 (0.6%)
Carbapenems + other penicillins	54 (0.4%)

A total of 18,344 antibiotic prescriptions including 69 different antibiotic agents were assessed at the surveyed points. The five most commonly prescribed antibiotic agents were ceftazidime (10.9%, *n* = 2,000), azithromycin (9.9%, *n* = 1,825), cefperazone/sulbactam (8.5%, *n* = 1,561), cefotaxime (7.6%, *n* = 1,388) and piperacillin/tazobactam (7.5%, *n* = 1,375). Meropenem accounted for 5.7% (*n* = 1,050) of all prescribed antibiotics. Amoxicillin and amoxicillin/clavulanic acid accounted for 0.9% (*n* = 160) and 5.0% (*n* = 919) of all antibiotic therapies, respectively. The most prescribed antibiotic ATC classifications were third-generation cephalosporins (41.9%, *n* = 7,679), penicillin/β-lactamase inhibitors (16.1%, *n* = 2,962), macrolides (12.1%, *n* = 2,214) and carbapenems (7.7%, *n* = 1,331). Third-generation cephalosporins were the most commonly prescribed antibiotics in 16 participating hospitals and all age groups. Piperacillin/tazobactam and amoxicillin/clavulanic acid were the most common penicillin/β-lactamase inhibitors combination, followed by ampicillin/sulbactam (*n* = 332), mezlocillin/sulbactam (*n* = 242), piperacillin/sulbactam (*n* = 59) and amoxicillin/sulbactam (*n* = 35).

For 14,757 inpatients in which antibiotic therapy was prescribed, 1,935 (13.1%) and 648 (4.4%) inpatients were prescribed with third-generation cephalosporins-based and carbapenem-based combination antibiotic therapy, respectively. The most common combination antibiotic therapy was third generation cephalosporins in combination with macrolides (Table 3).

**TABLE 3:** Antibiotic prescribing patterns based on diagnostic conditions on admission.

Diagnostic conditions on admission	Number of inpatients in which antibiotic therapy prescribed	Number (percentage) of inpatients in which combination antibiotic therapy prescribed
Lower respiratory tract infections	6,379	1,588 (23.6)
Non-infectious diseases	3,851	811 (21.1)
Central nervous system infections	816	137 (16.8)
Sepsis	600	225 (37.5)
Undifferentiated fever	564	125 (22.2)
Upper respiratory tract infections	465	53 (11.4)
Abdominal infection	410	175 (42.7)
Gastrointestinal infections	280	40 (14.3)
Other infectious diseases	254	59 (23.2)
Skin and soft tissue infections	189	16 (8.5)
Neonatal infection	186	63 (33.9)
Viral infection	161	14 (8.7)
Urogenital infections	123	8 (6.5)
Multiple infectious diagnoses	66	10 (15.2)
Bone and joint infection	64	5 (7.8)
Total	14,757	3,329 (22.6)

According to WHO AWaRe classification, Access, Watch and Reserve antibiotics accounted for 14.5% (*n* = 2,664), 70.4% (*n* = 12,915) and 1.5% (*n* = 266), respectively. The percentage of Access and Watch antibiotics ranged from 2.5 to 49.0% (median 17.2%, IQR 7.7–22.1%) and 30.1–89.4% (median 68.5%, IQR 49.5–80.4%) among 16 participating hospitals. Reserve group antibiotics were used in 12 tertiary hospitals and accounted for less than 5% in each hospital (median 0.6%, IQR 0.05–1.65%, range 0–4.4%). The percentage of Access antibiotics was less than 25% in all age groups while Watch antibiotics accounted for 55.7–74.3% among different age groups.

### Antibiotic Prescribing Patterns Based on Diagnostic Conditions on Admission

Of the 14,757 hospitalized children in which antibiotics were prescribed, the most common diagnostic condition on admission was lower respiratory tract infections (*n* = 6,379, 43.2%). Combination antibiotic therapy was common in patients with a diagnosis of lower respiratory tract infections (Table 3). Of note, 48.6% (*n* = 6,274) of Watch antibiotics and 34.8% (*n* = 464) of carbapenems were prescribed for patients with a diagnosis of lower respiratory tract infection on admission.

For children with a diagnosis of lower respiratory tract infection on admission, the percentage of WHO Access and Watch antibiotics were 11.4% (*n* = 928) and 73.8% (*n* = 6,018), respectively. Watch antibiotics were the most prescribed antibiotics (54.4–92.4%) among 11 hospitals. According to ATC classification, third-generation cephalosporins (46.6%) were the most prescribed antibiotics, followed by macrolides (20.5%) and penicillin/β-lactamase inhibitors (16.2%). The percentage of carbapenems was 5.5% and even >10% among three participating hospitals. The percentage of penicillins/β-lactamase inhibitors was >50% in one hospital (S3).

There were 850 antibiotic therapies prescribed for children with a diagnosis of sepsis on admission, in which, third-generation cephalosporins (34.8%) were the most prescribed antibiotics, followed by carbapenems (20.4%) and penicillins/β-lactamase inhibitors (14.1%). Besides, 300 (35.3%) antibiotic therapies were used for neonates, in which, meropenem (21.3%, *n* = 64) was the most prescribed antibiotic agent, followed by ampicillin/sulbactam (12.7%, *n* = 38) and ceftazidime (12.7%, *n* = 38).

### Antibiotic Prescribing Patterns by Ward Types

The percentage of patients in which antibiotics were prescribed varied by ward types, with a high percentage observed in PICUs (78.5%), pediatric medical wards (68.1%) and surgical wards (65.2%). The percentage of antibiotic prescribing (Table 4) and distribution of antibiotic ATC classes within different ward types varied by hospitals (Supplementary Figure S1).

**TABLE 4:** Antibiotic prescribing pattern by different ward types.

Hospitals	Number of patient prescribed antibiotics (percentage of patient prescribed antibiotics by ward, %)					
	Medical ward	Surgical ward	High risk ward	PICU	Neonatal ward	NICU
C1	600 (65.7)	2 (66.7)	201 (76.1)	40 (95.2)	183 (48.5)	
C2	362 (76.9)	62 (63.3)		13 (56.5)	138 (63.9)	
C3	231 (44.7)	322 (80.7)	188 (75.5)	158 (92.9)	231 (62.6)	115 (52.5)
C4	454 (80.9)	17 (94.4)			57 (62.6)	
C5	1,359 (68.9)	378 (58.1)	240 (59.3)	60 (76.9)	166 (52.7)	106 (76.3)
C6	378 (78.8)	52 (67.5)	2 (33.3)	20 (62.5)	175 (57.8)	
C7	1,089 (51.7)	484 (48.8)	171 (77.4)	188 (74.0)	396 (65.3)	110 (53.9)
C8	152 (70.4)	12 (35.3)		41 (69.5)	14 (25.9)	22 (32.8)
C10	1,962 (72.3)	602 (80.2)	297 (53.1)	54 (88.5)	267 (78.3)	153 (85.5)
C11	472 (81.0)	134 (87.0)	50 (49.0)	19 (63.3)	60 (58.3)	104 (48.6)
G1	207 (70.4)	50 (68.5)		43 (78.2)	29 (72.5)	
G3	173 (40.3)			22 (64.7)	27 (19.1)	
G5	82 (91.1)				98 (88.3)	
S1	314 (94.3)	10 (90.9)				
S3	71 (83.5)				16 (32.7)	
S4	395 (90.4)	12 (66.7)			45 (64.3)	
Total	12,197 (68.1)	3,278 (65.2)	1,806 (63.6)	838 (78.5)	3,186 (59.7)	1,022 (59.7)

The most obvious variation of antibiotic prescribing patterns was seen in neonatal wards among hospitals. The overall percentage of antibiotic prescriptions in neonatal wards were 59.7%, ranging from 19.1 to 88.3% (median 62.6%, IQR 50.6–64.8%) among hospitals. The percentages of Access and Watch antibiotics were 23.2 and 61.9% in neonatal wards, ranging from 2.8 to 56.7% (15.2%, IQR 8.8–36.7%) and 24.3–89.4% (61.9%, 52.1%–71.9%) among hospitals, respectively. In all, third-generation cephalosporins (39.4%) were the most commonly prescribed antibiotic category, followed by penicillins/β-lactamase inhibitors (26.0%) and carbapenems (9.5%). The percentage of carbapenems ranged from 1.1 to 24.2% (median 6.5%, IQR 1.2–9.9%) among hospitals.

For children hospitalized in PICUs of the 11 hospitals, lower respiratory tract infections (49.8%) were the most common diagnosis on admission. The percentage of children on antibiotic therapy ranged from 62.5 to 95.2% (median 74.0%, IQR 64.0–83.4%). Generally, third-generation cephalosporins (35.5%) and carbapenems (18.7%) were frequently prescribed antibiotic ATC classes in PICUs. In three hospitals, the percentage of carbapenems prescription was higher than 30%.

For children hospitalized in surgical wards of the 13 hospitals, the percentage of children on antibiotic therapy was 65.2%, ranging from 62.5 to 95.2%. For patients on antibiotic therapy, 40.1% had a diagnosis related to infectious conditions on admission, in which, the common diagnoses were abdominal infections (13.7%, *n* = 293), lower respiratory tract infections (5.7%, *n* = 122) and skin and soft tissue infections (5.4%, *n* = 116). Generally, third-generation cephalosporins (40.2%, *n* = 1,035) and second-generation cephalosporins (20.3%, *n* = 523) were the most prescribed antibiotic ATC classes in surgical wards.

For children hospitalized in high risk wards of seven hospitals, the percentage of children on antibiotic therapy was 63.6%, ranging from 33.3 to 77.4% (median 59.3%, IQR 51.1–75.8%). For children on antibiotic therapy, 66.1 and 41.8% had a diagnosis of either high risk disease or infectious disease on admission, respectively. Generally, third-generation cephalosporins (30.1%, *n* = 504), sulfonamids and trimethoprim (20.3%, *n* = 340) and carbapenems (14.9%, *n* = 249) were the frequently prescribed antibiotic ATC classes.

## Discussion

These repeated hospital-based point prevalence surveys of antibiotic use illustrate current situation regarding antibiotic prescription practice in pediatric inpatients in China. During the four time points cross-sectional surveys in 2019, the point prevalence of antibiotic use in pediatric wards was 66.1%, which was higher than the prevalence reported in high-income countries such as European countries (36.4%), Australia (46%) and the United States(42.6%) (Versporten et al., 2013; Osowicki et al., 2014), but was comparable to other Asian countries such as India (61.5%) and Vietnam (67.4%) (Thu et al., 2012; Gandra et al., 2017). For children in which antibiotics were prescribed, the percentage of inpatients <1 year (42.6%) was higher than that in developed countries (30%) (Osowicki et al., 2014). It is noteworthy that Watch group antibiotics accounted for 73.8% of antibiotic prescriptions and inappropriate combination antibiotic therapy was frequently prescribed in Chinese pediatric inpatients. Antibiotic stewardship needs to be strengthened to reduce unnecessary antibiotic use in Chinese children. On the other hand it is quite important to improve the uptake of pneumococcal conjugate vaccine, Haemophilus influenza type B conjugate vaccine, influenza vaccine and rotavirus vaccine in Chinese children, which are highly recommended by WHO to reduce the incidence of vaccine-preventable pneumonia and diarrhea whose treatment would require antimicrobial medicines or are often inappropriately treated with antibiotics (World Health Organization, 2012; World Health Organization, 2013a; World Health Organization, 2013b; World Health Organization, 2019a).

Our study noticed a significant variation in antibiotic use even across similar medical settings and within ward types in China. The point prevalence of antibiotic use ranged from 36.8 to 94.2% in 16 participating hospitals, from 19.1 to 88.3% in neonatal wards and from 35.3 to 94.4% in surgical wards. We also observed a substantial variation between hospitals in the use of Access, Watch, and Reserve antibiotics within ward types and diagnostic conditions. In the 15 neonatal wards, the percentage of Access and Watch group antibiotics ranged from 2.8 to 56.7% and from 24.3 to 89.4%, respectively. This situation in fact reflects inconsistency in antibiotic prescribing behavior and the poor compliance of the guidelines on common infectious diseases among pediatricians. Thus, there is an urgent need to implement standardized clinical pathways or guidelines of common infectious diseases and strengthening education to improve appropriate use of antibiotic.

Overall, Watch group antibiotics accounted for 70.5% of all antibiotic prescriptions and third-generation cephalosporins accounted for 41.9%. However, in most countries, Watch group antibiotic use in hospitalized children was less than 50% (Hsia et al., 2019a), and third-generation cephalosporins use in hospitalized children was even below 10% in West Europe, Australia and North America (Versporten et al., 2016). According to WHO surveillance data in 2020, the prevalence of third-generation cephalosporins resistance in *Escherichia coli* (*E.coli*) and *Klebsiella pneumoniae* among blood stream infection was 36.0 and 56.7%, respectively (World Health Organization, 2020). In the United States, the prevalence of ESBL-producing Enterobacterales causing bloodstream infections in pediatric patients is 5.2% (Sader et al., 2020). In low- and middle-income countries, the prevalence of third-generation cephalosporins resistance in *E. coli* and *Klebsiella pneumoniae* is 27 and 74%, respectively (Droz et al., 2019). In China, the prevalence of *ESBL*-producing Enterobacteriaceae is among the highest in the world (>50%) (Hu et al., 2018; National Health Commission of the People’s Republic of China, 2018b), which is partly related to overuse of third-generation cephalosporins since the late 1990s. In terms of the diagnoses on admission, most of prescribed antibiotics were used for the treatment of common community-acquired respiratory infections in pediatric inpatients. Thus, it is feasible to improve the overuse of third-generation cephalosporins in Chinese children through intensive antimicrobial stewardship programs.

An additional noteworthy problem was the frequent use of carbapenems in Chinese pediatric inpatients. Carbapenems accounted for 7.4% of all antibiotic prescriptions and even up to 9.9% among neonates, which was significantly higher than that reported in India (4.5%) and Australia (3.2%) (Osowicki et al., 2014; Gandra et al., 2017). For children hospitalized in PICUs, carbapenems prescriptions accounted for 18.7% and even more than 30% in some facilities. The frequent use of carbapenems is mainly driven by high prevalence of ESBL-producing Enterobacteriaceae and strict restriction of aminoglycosides use for children in China (National Health Commission of the People’s Republic of China, 2018a). On the other hand, carbapenems are also misused for either non-severe bacterial infections or resistant-bacteria colonization for a clinical concern about “just in case” in pediatric practice. In the United States, prevalence of carbapenem resistance in Enterobacteriaceae is only 0.5% in pediatric patients (Sader et al., 2020). In the European countries, the prevalence of carbapenem resistance in *E. coli* and *Klebsiella pneumoniae* is 0.6 and 6.5% in pediatric patients (Bielicki et al., 2015). So far, increasingly high prevalence of carbapenem resistance in Enterobacteriaceae and *Acinetobacter baumannii* has been a big clinical challenge in Chinese pediatric patients (National Health Commission of the People’s Republic of China, 2018b). The Chinese health authority issued expert consensus on clinical use of carbapenems in 2018, in which, empirical and target carbapenems therapies should be prescribed based on strict clinical indications and microbiological evidence, meanwhile, carbapenem prescriptions need to be documented and supervised (National Health Commission of the People’s Republic of China, 2018a). Improving the use of carbapenems should be a key priority in pediatric antimicrobial stewardship programs in China.

The study has several strengths. First, we applied comprehensive and child-appropriate indications based on WHO AWaRe methodology to assess the appropriateness of pediatric antibiotic prescribing. Second, we adopted standardized methodology of point prevalence survey and a large sample size to reduce the bias of surveillance data. Third, we compared the variation in antibiotic prescribing practice among hospitals and by ward types.

The study also has a few of limitations. First, the appropriateness of antibiotic prescribing could not be determined based on the detailed patient characteristics, indication of antibiotic use, severity of infection, duration of therapy, or microbiology and antimicrobial susceptibility results. Second, most of participating hospitals in this study were provincial or municipal tertiary hospitals on a voluntary basis, thus, our results cannot fully represent the current situation of antibiotic use in secondary hospitals and county hospitals in China. However, our surveillance data remain representative and reflected the objective situation and issues on antibiotic use in Chinese pediatric inpatients because the majority of pediatric inpatients are hospitalized in the tertiary hospitals in China. The results provided useful information for pediatricians to improve clinical practice and for policy-makers to strengthen the pediatric antimicrobial stewardship in the future.

## Materials and Methods

This study is designed prospectively to retrieve point prevalence surveys (PPS) of antibiotic use in hospitalized children in China referring to WHO Methodology of Point Prevalence Survey on Antibiotic Use in Hospitals (World Health Organization, 2018b). Sixteen public hospitals from the pediatric subgroup of the Chinese Society of Infectious Diseases participated in this study on a voluntary basis. Cross-sectional hospital-based PPSs were conducted in all pediatric and neonatal wards of the participating hospitals on the second Monday of March, June, September and December in 2019 (11 March, 10 June, 9 September and 9 December). Among the 16 hospitals, 13 were tertiary care hospitals and three were secondary care hospitals; four were general hospitals and 12 were children’s specialized hospitals (including seven maternal and children’s hospitals) (Table 5).

**TABLE 5:** General information of participating hospitals.

Code	Hospital	Type	Level	Ward types^a^	Total beds
C1	West China second hospital, sichuan University	Maternal and children’s	Tertiary	M, S, P, n, H	565
C2	Xiamen Children’s hospital	Children’s	Tertiary	M, S, P, n	1,000
C3	Children’s hospital of fudan University	Children’s	Tertiary	M, S, P, N, n, H	686
C4	Hangzhou Children’s hospital	Children’s	Tertiary	M, S, n	350
C5	Children’s hospital of jiangxi province	Children’s	Tertiary	M, S, P, N, n, H	1,200
C6	Hainan Children’s hospital	Children’s	Tertiary	M, S, P, n, H	220
C7	Hunan Children’s hospital	Children’s	Tertiary	M, S, P, N, n, H	1,800
C8	Gansu provincial maternity and child-care hospital	Maternal and children’s	Tertiary	M, S, P, N, n	1,082
C10	Children’s hospital affiliated to zhengzhou University	Children’s	Tertiary	M, S, P, N, n, H	2,227
C11	Qingdao women and children hospital	Maternal and children’s	Tertiary	M, S, P, N, n, H	1,032
G1	The fourth hospital of baotou	General	Tertiary	M, S, P, n	156
G3	The first affiliated hospital of guangxi medical University	General	Tertiary	M, P, n	202
G5	Zhongnan hospital of wuhan University	General	Tertiary	M, n	55
S1	Chengdu children special hospital	Children’s	Secondary	M, S	130
S3	Lishui maternal and child health care hospital	Maternal and children’s	Secondary	M, n	
S4	People’s hospital of zhecheng county	General	Secondary	M, S, n	186

^a^ H, high risk ward; M, pediatric medical ward; N, neonatal intensive care unit; n, neonatal ward; P, pediatric intensive care unit; S, pediatric surgical ward.

All neonates and pediatric patients were included in the survey if they met the following criteria: younger than 16 years of age, present in the ward at 8:00 a.m. on the day of the survey and having been hospitalized in specialized pediatric wards for at least 24 h.

All data were retrieved via the hospital information system and finally reviewed by two infectious diseases specialists to exclude invalid or incomplete records. The exclusion criteria for sampled patients were as the following: 1) patients hospitalized in emergency observational wards, rehabilitation wards, and daycare wards; 2) hospitalized patients with incomplete information about patient’s demographics, diagnoses or medication.

Patient demographic data were collected for all surveyed patients, irrespective of antibiotic treatment. Antibiotic data were collected for patients on antibiotic therapy and included information on the antibiotics prescribed, such as the type of antibiotics, route of administration and the diagnoses on admission. Reason for prescribing antibiotics was not routinely available via hospital information systems. Therefore, we assessed the antibiotic prescribing roughly based on admission diagnoses.

In this study, “pediatric wards” were grouped into pediatric medical ward, pediatric surgical ward, pediatric intensive care unit (PICU), neonatal ward, neonatal intensive care unit (NICU) and high-risk ward. “High-risk wards” included hematology and oncology wards. “Antibiotics” included antibacterial agents for systemic use listed in the Anatomical Therapeutic Chemical (ATC) class J01 and orally administered metronidazole (P01AB01). In addition, cefathiamidine (J01DBX) and kitasamycin (J01FAX) which are not included in the WHO ATC classification were coded according to the pharmacological characteristics and the classification rules of the ATC guideline (World Health Organization, 1990). Antibiotics were grouped into 10 classes according to the ATC system (Table 6). The term “antibiotic prescription” was defined as the use of one antibiotic agent by one route of administration. Diagnoses on admission were divided into 15 diagnostic conditions on the clinical ground: upper respiratory infections; lower respiratory infections; gastrointestinal infections; abdominal infections; central nervous system infections; sepsis; bone and joint infections; undifferentiated fever; skin and soft tissue infections; urogenital infections; viral infections; neonatal infections; other infectious conditions; multiple infectious conditions; non-infectious conditions.

**TABLE 6:** Antibiotic ATC classification.

Antibiotic categories	ATC
Penicillins/β-lactamase inhibitors	J01CR01, J01CR02, J01CR03, J01CR05
Other penicillins	J01C (excluding J01CR01, J01CR02, J01CR03, J01CR05)
Second generation cephalosporins	J01DC
Third generation cephalosporins	J01DD
Fourth generation cephalosporins	J01DE
Carbapenems	J01DH
Macrolides	J01FA
Sulfonamids and trimethoprim	J01E
Other antibacterials	J01X
Others	J01A Tetracycline
	J01DB First generation cephalosporins
	J01DF Monobactams
	J01FF Lincosamides
	J01FG Streptogramins
	J01G Aminoglycoside
	J01M quinolone
	P01AB01 Metronidazole

The following antibiotic prescribing indicators were calculated:

1. the percentage of hospitalized children in which >1 antibiotic was prescribed was calculated by dividing the number of hospitalized children prescribed ≥1 antibiotics by the total number of hospitalized children and multiplying by 100;
2. the percentage of hospitalized children in which ≥1 intravenous antibiotic was prescribed was calculated by dividing the number of hospitalized children prescribed ≥1 intravenous antibiotics by the total number of hospitalized children prescribed antibiotics and multiplying by 100;
3. the percentage of hospitalized children in which combination antibiotic therapy was prescribed was calculated by dividing the number of hospitalized children prescribed >1 antibiotics by the total number of hospitalized children prescribed ≥1 antibiotic and multiplying by 100;
4. the percentage of antibiotics prescribed in each ATC class or WHO AWaRe category was calculated by dividing the number of antibiotic prescriptions prescribed in a certain ATC class or AWaRe category by the total number of antibiotics prescriptions prescribed and multiplying by 100.

These indicators were calculated separately for each hospital, ward type, diagnostic condition and age group (0 to <1 month, 1 month to <1 year, 1 to <3 years, 3 to <7 years, 7 to <12 years and 12 to <16 years).

All data were exported into an Excel spreadsheet. Descriptive analyses were conducted by using Excel (Microsoft, Redmond, United States), Access (Microsoft, Redmond, United States) and GraphpadPrism (GraphPad Software, San Diego, CA).

Ethics approval was received for all participating hospitals from their respective institutional human research ethics committees. Informed consent from patients was not required by the ethics committee because there was no contact with patients and all data were de-identified.

## Data Availability

The raw data supporting the conclusions of this article will be made available by the authors, without undue reservation.
