# Supplementary material for: Antibiotic Use Among Hospitalized Children and Neonates in China: Results From Quarterly Point Prevalence Surveys in 2019
Source: Front Pharmacol. 2021 Mar 29;12:601561. doi: 10.3389/fphar.2021.601561 (PMC8039455; doi:10.3389/fphar.2021.601561)
Supplement: Supplementary file 1 [file datasheet1.pdf]

Supplementary Material

1.1 Supplementary Figure

Supplementray Figure1. Distribution of antibiotic ATC classes in different ward types

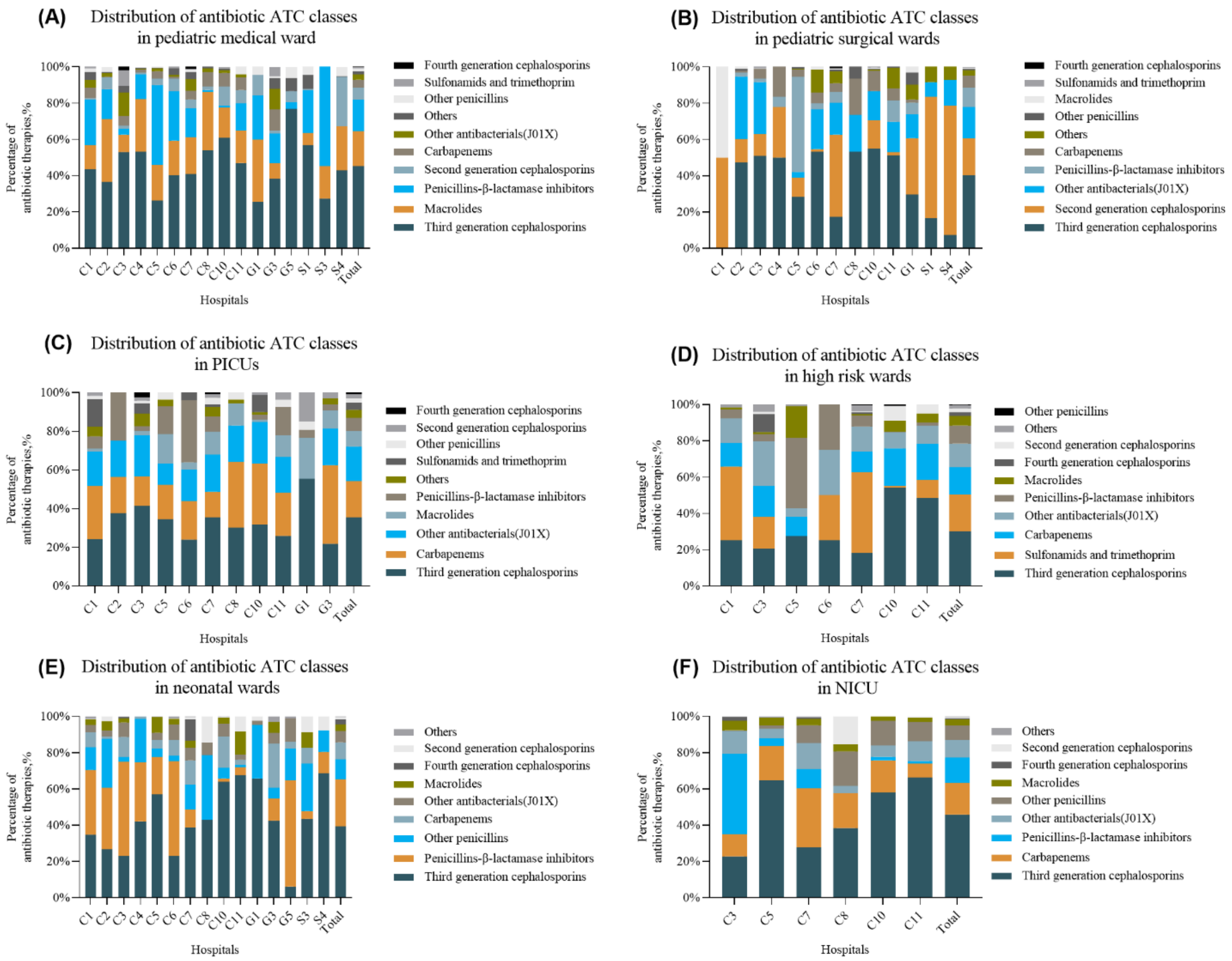

Supplementary Figure 1. Distribution of antibiotic ATC classes within different ward types varied by hospitals.
